# Возможности и перспективы формирования генетической защиты мягкой пшеницы от стеблевой ржавчины в Западной Сибири

**DOI:** 10.18699/VJ20.679

**Published:** 2020-12

**Authors:** V.N. Kelbin, E.S. Skolotneva, E.A. Salina

**Affiliations:** Institute of Cytology and Genetics of Siberian Branch of the Russian Academy of Sciences, Novosibirsk, Russia; Institute of Cytology and Genetics of Siberian Branch of the Russian Academy of Sciences, Novosibirsk, Russia; Institute of Cytology and Genetics of Siberian Branch of the Russian Academy of Sciences, Novosibirsk, Russia

**Keywords:** bread wheat, stem rust, resistance genes, marker-assisted selection, phytopathological test, мягкая пшеница, стеблевая ржавчина, гены устойчивости, маркер-ориентированная селекция, фитопатологическое тестирование

## Abstract

Современные исследования проблемы устойчивости мягкой пшеницы к стеблевой ржавчине включают два основных направления: оценку устойчивости коллекций мягкой пшеницы к заболеванию с помощью молекулярных маркеров к известным генам устойчивости в дополнение к полевому скринингу материала и лабораторным тестам к образцам различных популяций гриба; поиск источников и доноров новых генов и генных локусов, в том числе среди культурных и дикорастущих родичей пшеницы. Для достижения адекватного генетического контроля заболевания важен интегральный подход, включающий как данные об источниках устойчивости, так и актуальные сведения о действующих в регионе патогенных популяциях, их расовом составе и динамике генов вирулентности. Результаты анализа экспериментальных данных полевого скрининга устойчивости к стеблевой ржавчине сортов мягкой пшеницы из коллекции питомников CIMMYT в условиях Омской и Новосибирской областей, а также лабораторного тестирования образцов инфекции на международном наборе пшеничных линий-дифференциаторов позволяют предполагать, что на территории Западной Сибири и Алтайского края существует обособленная, «азиатская», популяция Puccinia graminis f. sp. tritici. При этом практический интерес для современных программ опережающей селекции пшеницы на иммунитет к стеблевой ржавчине в условиях Западной Сибири представляют гены устойчивости Sr2, Sr6Ai#2, Sr24, Sr25, Sr26, Sr31, Sr39, Sr40, Sr44 и Sr57. В настоящем обзоре проанализированы источники генов, сохраняющих эффективность к западносибирской популяции P. graminis, с целью упрощения первичного этапа отбора селекционного материала для создания устойчивого генотипа путем пирамидирования генов. Описаны основные требования, предъявляемые к фитопатологическому тестированию селекционного материала. Составлен список молекулярных маркеров к указанным генам устойчивости – как широко применяющихся в маркер-ориентированной селекции, так и требующих верификации.

## Abstract (en)

Current studies on bread wheat resistance to stem rust have two main subjects: complex analysis for resistance of bread wheat germplasm using molecular markers, field screening and laboratory tests against samples of different fungal populations, and searching for sources and donors of new genes and gene loci, including cultivated and wild relatives of wheat. To achieve adequate genetic control of the disease, an integral approach is important, incorporating both data on sources of resistance and relevant information on pathogenic populations existing in the region, their race composition and dynamics of virulence genes. The analysis of experimental data on field screening of bread wheat varieties from the CIMMYT nursery germplasm for stem rust resistance in the Omsk and Novosibirsk regions, together with laboratory testing of infection samples on the international set of wheat differential lines, suggests that a separate “Asian” population of Puccinia graminis f. sp. tritici exists in Western Siberia and the Altai Territory. Wheat resistance genes Sr2, Sr6Ai#2, Sr24, Sr25, Sr26, Sr31, Sr39, Sr40, Sr44, and Sr57 are of practical interest for advanced wheat breeding programs for stem rust immunity in Western Siberia. This review provides an analysis of the gene sources that remain effective against the West Siberian population of P. graminis, in order to facilitate the initial stage of selection of breeding material to develop a stable genotype by gene pyramiding. The basic requirements for conducting a phytopathological test of breeding material are presented. A list of molecular markers for the mentioned resistance genes, both widely used in marker-assisted selection and requiring verification, has been compiled.

## Введение

До конца прошлого века значимость болезни, вызванной биотрофным грибом P. graminis, была снижена повсеместно успешными программами селекции на иммунитет. Однако в последнее время для регионов возделывания мягкой пшеницы характерно ухудшение фитопатологической обстановки, связанной со стеблевой ржавчиной: Северной и Южной Америки (Singh R.P. et al., 2016), Восточной Африки (Patpour et al., 2016), Австралии (Addai et al., 2018), Западной Европы (Lewis et al., 2018) и Казахстана (Рсалиев А.С., Рсалиев Ш.С., 2018). Развитию патогена главным образом способствуют благоприятные климатические условия (Shamanin et al., 2013; Morgounov et al., 2014). Причиной массовых эпифитотий пшеницы в Уганде (1998–1999), Кении и Эфиопии (2005–2006), Йемене (2006), Иране (2007) и Пакистане (2009) стало появление и быстрое распространение новой агрессивной расы Ug99 (Prasad et al., 2016). Существует реальная угроза поражения расой и ее модификациями, так называемым семейством рас Ug99 (TTKSK, TTKSF, TTKST, TTTSK, TTKSP, PTKSK, PTKST, TTKSF+, TTKTT, TTKTK и TTHSK), производственных посевов пшеницы в Казахстане и Западной Сибири, на Урале и в других регионах Российской Федерации (Шаманин и др., 2015). В этой связи проводят оценку устойчивости существующих коллекций мягкой яровой пшеницы как к местным популяциям патогенов, так и популяциям патогенов, распространенных в соседних регионах.

Выявление источников устойчивости является повсеместной задачей. В результате скрининга коллекций мягкой яровой пшеницы в Индии (Sharma et al., 2015) и Эфиопии (Soresa, 2018) получены сходные данные: доля устойчивых генотипов к местному возбудителю стеблевой ржавчины, а также Ug99 в коллекциях оказалась минимальной. В России над созданием исходного материала и сортов с устойчивостью к стеблевой ржавчине успешно работают научный коллектив Федерального исследовательского центра «Немчиновка» (Московская область) под руководством д-ра биол. наук И.Ф. Лапочкиной и специалисты Всероссийского научно-исследовательского института защиты растений (Санкт-Петербург, Пушкин). На базе исходного материала, выделенного из коллекции генетических ресурсов растений ВИР и коллекции «Арсенал», созданы линии озимой пшеницы, устойчивые к стеблевой ржавчине в условиях Нечерноземной зоны России. Значительные результаты по генотипированию сортов яровой мягкой пшеницы, а также интрогрессивных линий с генетическим материалом от чужеродных видов (Aegilops speltoides, Agropyron elongatum, Aegilops triuncialis, Secale cereale) селекции Федерального аграрного научного центра Юго-Востока получены канд. биол. наук О.А. Барановой. У исследуемых интрогрессивных линий постулированы гены Sr31/Lr26, Sr25/Lr19, Sr28, Sr57/ Lr34 и Sr38/Lr37. Сочетание генов Sr31/Lr26 и Sr25/Lr19 идентифицировано у 26.3 % линий и сортов, возделываемых на территории Поволжья (Baranova et al., 2019).

Для адекватного генетического контроля заболеваний важен интегральный подход, включающий как данные об источниках устойчивости, так и актуальные сведения о действующих в регионе патогенных популяциях, их расовом составе и динамике генов вирулентности. Целью данного обзора был интегральный анализ фитосанитарной ситуации по стеблевой ржавчине в Западной Сибири в отношении перспективы формирования генетической защиты мягкой пшеницы.

## Эффективность известных генов Sr в условиях Западно-Сибирского региона

С 2007–2009 гг. значение стеблевой ржавчины в фитопатогенном комплексе пшеницы Западной Сибири возросло (Сочалова, Лихенко, 2015). Путем сравнения расового состава образцов инфекции из Омска и Новосибирска выяснено, что первичной зоной формирования инокулюма P. graminis является Омская область (Сколотнева и др., 2020). Комплексное исследование культивируемых на территории Западной Сибири сортов мягкой пшеницы показало, что большинство из них восприимчивы к заболеванию, а остальные защищены небольшим количеством генов устойчивости: Sr25, Sr31, Sr36, Sr6Ai, Sr6Ai#2 (Shamanin et al., 2016; Leonova et al., 2020). При этом оценка коллекции мягкой яровой пшеницы Омского государственного аграрного университета на естественном инфекционном фоне лесостепи Западной Сибири продемонстрировала, что только 10 % сортов коллекции устойчивы к местному патогену. По данным скрининга коллекции к Ug99 в Кении, доля устойчивых к агрессивной расе сортов также не превышает 10 % (Шаманин и др., 2015).

Изучение образцов стеблевой ржавчины Западной Сибири в последние несколько десятилетий выявило изменчивость вирулентности к генам устойчивости пшеницы Sr6, Sr7b, Sr8a, Sr9e, Sr11, Sr21, Sr30 и Sr36 (Сколотнева и др., 2020). Результаты полевого скрининга набора сортов с генами Sr из коллекции питомников CIMMYT в условиях Омской и Новосибирской областей, а также лабораторного анализа образцов инфекции на международном наборе пшеничных линий-дифференциаторов позволяют предположить, что на территории Западной Сибири и Алтайского края представлена обособленная (так называемая азиатская) популяция P. graminis (Shamanin et al., 2020). Она отличается высокой вирулентностью к генам Sr5, Sr9a, Sr9b, Sr9d, Sr9g, Sr10, Sr17, Sr38 и SrMcN. Интерес для современных программ опережающей селекции на иммунитет к стеблевой ржавчине в условиях Западной Сибири представляют гены устойчивости Sr2, Sr6Ai#2, Sr24, Sr25, Sr26, Sr31, Sr39, Sr40, Sr44, Sr57, для которых показано преобладание авирулентных клонов в местных субпопуляциях гриба (Shamanin et al., 2016; Skolotneva et al., 2018; Сколотнева и др., 2020).

## Присутствие генов Sr в мировом селекционном материале

Sr2 является одним из наиболее важных генов в современной селекции на иммунитет к стеблевой ржавчине, так как обеспечивает длительную устойчивость взрослых растений (McIntosh, 1988; Roelfs, 1988; Simmonds, Rajaram, 1988). В 1979 г. описано замедленное течение патогенеза P. graminis на растениях, несущих ген Sr2, что позволило отнести обеспечиваемую им устойчивость к неспецифическому типу (Hare, McIntosh, 1979). Физиологическим маркером гена Sr2 является характерное почернение чешуй колоса (pseudo black chaff). Кроме того, ассоциированным признаком, проявляющимся при тепличной температуре выше 22 °C, является хлороз листьев на стадии проростков (Brown, 1993). За исключением Канады ген Sr2 обеспечивает эффективную устойчивость повсеместно с момента его введения в гексаплоидную пшеницу в 1920-х гг. (McFadden, 1930). Во время эпифитотий в Северной Америке в 1950-х гг. сорта пшеницы Regent, Renown и Redman с геном Sr2 показали умеренную восприимчивость. Сорта Pavon 76 и Buck Buck с комбинацией генов Sr2 и Sr23, испытанные в условиях Западной Сибири, продемонстрировали устойчивость к локальной популяции стеблевой ржавчины (Шаманин и др., 2015). Тестирование на естественном инфекционном фоне Нечерноземной зоны России новых линий озимой пшеницы, созданных на основе коллекций ВИР и «Арсенал», позволило выявить эффективное сочетание двух и более генов ювенильной устойчивости (Sr22, Sr32, Sr39 и Sr40) с геном Sr2 (Лапочкина и др., 2018). В настоящий момент база данных GRIS (http://wheatpedigree.net/) содержит 1762 наименования сортов и линий пшеницы, которые несут ген устойчивости Sr2.

Sr6Ai#2 находится в составе группы генов Lr6Ai#2/ Sr6Ai#2/Pm6Ai#2, обеспечивающих устойчивость к комплексу листостебельных заболеваний пшеницы, и расположен в хромосоме 6Ai#2, которая интрогрессирована в мягкую пшеницу от Thinopyrum intermedium. Цитогенетическое исследование сортов Тулайковская-5, Тулайковская-10 и Тулайковская-100, имеющих многолетнюю историю культивирования в различных регионах России, показало, что хромосома 6Ai#2 сохранила свою целостность в данных сортах (Salina et al., 2015). Среди отечественного материала присутствие гена Sr6Ai#2 продемонстрировано для линий и сортов cаратовской и самарской селекции в сочетании с генами Sr31 и Sr25 (Shamanin et al., 2016).

Sr24, ген устойчивости к стеблевой ржавчине, вместе с Lr24, геном устойчивости к бурой ржавчине, перенесен в пшеницу от Ag. elongatum. Известна спонтанная транслокация (3Ag) в хромосоме 3DL, описанная в сорте Agent (Smith et al., 1968). Получены рекомбинантные линии, в которых удалось нарушить сцепление генов устойчивости и признака красной пигментации зерна (Sears, 1973), что позволило интрогрессировать ген Sr24 в белозерную пшеницу.

Комплекс генов Lr24/Sr24 обеспечивает эффективную защиту от основных ржавчинных заболеваний пшеницы по всему миру за исключением Южной Африки (Roux, 1985; Pretorius et al., 2010), Индии (Bhardwaj et al., 2010; Manjunatha et al., 2015) и Кении (Jin et al., 2008), где выявлены новые вирулентные расы стеблевой ржавчины. Вирулентные к гену Lr24 клоны возбудителя бурой ржавчины зарегистрированы в Австралии, Чехии, Иране и США (Park et al., 2002; Kolmer, 2019; Hanzalova et al., 2020; Nemati et al., 2020). В России, в Омской области, недавно выявлены расы P. graminis, вирулентные к гену Sr24 (Shamanin et al., 2020; Skolotneva et al., 2020). В базу данных GRIS загружена информация о 903 сортах и линиях пшеницы, которые несут ген устойчивости Sr24.

Sr25, ген расоспецифической устойчивости, перенесен в длинные плечи хромосом 7D и 7A от Thinopyrum ponticum с комплексом генов резистентности к бурой ржавчине Lr19 и геном, контролирующим желтую окраску муки (Friebe et al., 1996; Zhang et al., 2005).

Ген Sr25 введен в австралийские сорта пшеницы и использован в программе селекции пшеницы CIMMYT, где одним из его основных источников является сорт Wheatear (Bariana et al., 2007). Показана повсеместная эффективность гена Sr25 по отношению к расам семейства Ug99 (Singh R.P. et al., 2011). Среди отечественного материала присутствие гена Sr25 отмечено для линий и сортов cаратовской, самарской и омской селекции (Shamanin et al., 2016). База данных GRIS в настоящий момент содержит 92 наименования сортов и линий пшеницы, которые несут этот ген.

Sr26, эффективный в отношении семейства рас Ug99, перенесен от Ag. elongatum в дистальную область длинного плеча хромосомы 6А (Knott, 1961). Транслокация 6AS.6AL-6Ae#1L заметно влияла на урожайность созданных линий и сортов, потери составляли 9 %. Ген Sr26 использован в качестве источника устойчивости к стеблевой ржавчине, в основном в Австралии, где был создан сорт Eagle. В настоящее время созданы новые линии с укороченными фрагментами транслокаций, показавшие высокие показатели качества и урожайности (Dundas et al., 2007). Таким образом, исторически сложившаяся низкая частота Sr26 среди современных сортов и создание донорских линий с короткими чужеродными сегментами делает ген Sr26 наиболее подходящим для использования в селекционных программах. В базу данных GRIS загружена информация о 61 сорте и линии пшеницы, которые несут ген Sr26.

Sr31 унаследован от ржи сорта Petkus в составе транслокации 1BL.1RS вместе с генами, контролирующими устойчивость растений к другим грибным патогенам: бурой ржавчине (Lr26 ), желтой ржавчине (Yr9) и мучнистой росе (Pm8) (Singh N.K. et al., 1990; McIntosh et al., 1993). Пшенично-ржаная транслокация нашла интенсивное применение в селекционных программах различных регионов мира, в которых родительскими формами были отечественные сорта пшеницы Kavkaz и Aurora, носители этой транслокации (Rabinovich, 1998; Zhou et al., 2003; Schlegel, 2010). За последние сорок лет селекционеры широко использовали транслокацию 1BL.1RS для улучшения агрономических характеристик мягкой пшеницы, особенно урожайности зерна. Ген Sr31 присутствует во многих сортах, районируемых в России, Европе, Китае и США, а также в селекционном материале, распространяемом программой CIMMYT, например в сортах Bobwhite и Veery (Carver, Rayburn, 1994; Lelley et al., 2004; Shamanin et al., 2016). В базе данных GRIS содержится 1119 наименований сортов и линий пшеницы, которые несут пшенично-ржаную транслокацию 1BL.1RS.

В большинстве регионов низких широт ген Sr31 утратил актуальность в связи с распространением из стран СевероВосточной Африки рас семейства Ug99, вирулентных к этому гену (Singh R.P. et al., 2006), однако Sr31 остается эффективным на территории России, в том числе в Западно-Сибирском регионе (Волкова и др., 2014; Сколотнева и др., 2020).

Sr39 обеспечивает устойчивость ко всем известным в настоящее время патотипам P. graminis, в том числе к семейству рас Ug99 (Mago et al., 2009). Ген перенесен в хромосому 2B сорта Marquis из генома Ae. speltoides в составе большой транслокации вместе с геном устойчивости к бурой ржавчине Lr35 (Kerber, Dyck, 1990). Разные авторы сообщают как об отрицательном, так и о положительном влиянии на хозяйственно важные характеристики транслокационных линий. Например, показано увеличение гигроскопичности муки у южноафриканской линии пшеницы Karee*6/RL6082 с геном Sr39 (Labuschagne et al., 2002). Разработаны транслокационные линии с уменьшенными чужеродными сегментами, которые обеспечивают групповую устойчивость к ржавчинным болезням за счет генов Sr39 и Lr35 (рекомбинант #247) (Mago et al., 2009). Всего шесть линий с геном Sr39 представлено в базе данных GRIS, из которых четыре имеют канадское происхождение, а линия Line-292 является результатом отечественной селекции.

Sr40 (SrA), обеспечивающий высокий уровень ювенильной и возрастной устойчивости к семейству рас Ug99, интрогрессирован в пшеницу от Triticum timopheevii ssp. armeniacum в составе транслокации T2BL/2G#2S (Friebe et al., 1996; Wu et al., 2009). После скрининга селекционного материала на устойчивость к расам из семейства Ug99 в условиях сильной инфекционной нагрузки кенийских полевых питомников ген Sr40 рекомендован для использования в коммерческих сортах пшеницы (Jin et al., 2007). В настоящий момент база данных GRIS содержит 9 наименований сортов и линий пшеницы, которые несут ген Sr40.

Sr44 (SrAgi) перенесен в геном мягкой пшеницы от Thinopyrum intermedium в составе транслокации 7Ai#1S (Cauderon et al., 1973; Friebe et al., 1996). Как и гены Sr25, Sr26, Sr39 и Sr40, ген Sr44 способен эффективно защищать растения от поражения расами семейства Ug99 (Liu W. et al., 2013). В базу данных GRIS внесена информация о четырех сортах мягкой пшеницы, которые несут ген Sr44. В их числе сорт ростовской селекции Донская полукарликовая.

Sr57 (Lr34/Yr18/Pm38/Bdv1) – плейотропный ген, обеспечивающий неспецифическую устойчивость к биотрофным патогенам, в том числе стеблевой ржавчине, локали зован в хромосоме 7DS (Krattinger et al., 2009; Lagudah et al., 2009; Dakouri et al., 2010). Эффект защитной реакции генотипов с Sr57 на различных инфекционных фонах описан как возрастная устойчивость (adult plant resistance) (McIntosh et al., 2010). Источником гена Sr57 являются стародавние итальянские сорта мягкой пшеницы Ardito и Mentana, созданные в 1900-х гг., при этом он сохраняет эффективность в течение столетия (Kolmer et al., 2008). Среди репрезентативной коллекции западноевропейских сортов ген обнаружен только у сорта Кавказ, однако Sr57 широко распространен среди американских, канадских и австралийских сортов (Kolmer et al., 2008), а также среди украинских сортов озимой мягкой пшеницы (Karelov et al., 2011). Большинство сортов омской и казахской селекции с геном Sr57 принадлежат к группам Ekada и Fiton соответственно (Shamanin et al., 2016). В последнее время неспецифический ген Sr57 с успехом применяют для создания генотипов с длительной устойчивостью методом пирамидирования генов. База данных GRIS содержит 2171 наименование сортов и линий пшеницы, которые несут ген Sr57.

## Методы постулирования генов Sr

До разработки первых молекулярных маркеров присутствие в селекционном материале генов устойчивости определяли эмпирически с помощью фитопатологического постулирования в соответствии с законом «ген на ген» – взаимодействия хозяина и патогена (Flor, 1947). Суть постулата заключается в том, что каждому гену устойчивости или восприимчивости растения-хозяина соответствует определенный комплементарный ген вирулентности или авирулентности паразита. К использованию фитопатологического постулирования (фитопатологического тестирования) прибегают до сих пор как к альтернативному подходу, позволяющему верифицировать молекулярные маркеры. Кроме того, данный метод остается единственно возможным в случае идентификации генов, для которых ДНК-маркеры не разработаны. Обязательным условием фитопатологического тестирования селекционного материала является поддержание в лаборатории рабочей коллекции чистых линий гриба, обладающих противоположными аллелями генов Avr, в данном случае изолятов P. graminis, вирулентных и авирулентных к искомому гену устойчивости Sr. При этом исследование проводят на ювенильной стадии растений, оценивая и сравнивая реакции (инфекционные типы) на заражение системами изолятов гриба (McVey, Roelfs, 1975). Проявление высокой восприимчивости (инфекционные типы 3 и 4 по балльной шкале, разработанной E.C. Stakman и коллегами (1962)) у тестируемой линии свидетельствует об отсутствии в генотипе генов устойчивости, к которым изолят P. graminis авирулентен. Так, восприимчивый тип реакции на заражение изолятом, авирулентным к Sr5, сообщает о том, что тестируемая линия не несет ген Sr5. Присутствие гена было бы сопряжено с устойчивостью (инфекционные типы 0, 1 и 2). 

Необходимо учитывать подготовительный этап подбора контрольных изолятов или патотипов P. graminis с определенной вирулентностью. Их используют, чтобы исключить присутствие гена устойчивости у исследуемого сорта, если сорт восприимчив хотя бы к одному патотипу, авирулентному к гену; предположить наличие гена на основании совпадения реакции совместимости патотипов с изучаемым сортом и линией, имеющей ген устойчивости. Методы закладки опытов и заражения проростков пшеницы стеблевой ржавчиной, в том числе уход за опытными растениями и оптимальный температурно-временной режим в период экспериментов, подробно описаны в разных публикациях (Jin et al., 2007; Li et al., 2016; Рсалиев А.С., Рсалиев Ш.С., 2018; Flath et al., 2018).

Выявленный эмпирическим путем ген должен быть обязательно подтвержден дополнительными исследованиями, такими как генетический и/или цитогенетический анализы. В настоящее время использование молекулярных маркеров является достойной альтернативой, позволяющей сократить время анализа с помощью оптимизированных протоколов. Список маркеров к генам Sr2, Sr6Ai#2, Sr24, Sr25, Sr26, Sr30, Sr31, Sr39, Sr40, Sr44, Sr57, сохраняющих эффективность к западносибирской популяции P. graminis и верифицированных в отечественных лабораториях, приведен в таблице. Для гена Sr40 в литературе предложены ДНК-маркеры различного типа, чаще всего микросателлитные (SSR) (Bernardo et al., 2013). Однако SSR-маркеры требуют оценки степени достоверности выявления гена на широком генетическом материале.

**Table 1.**
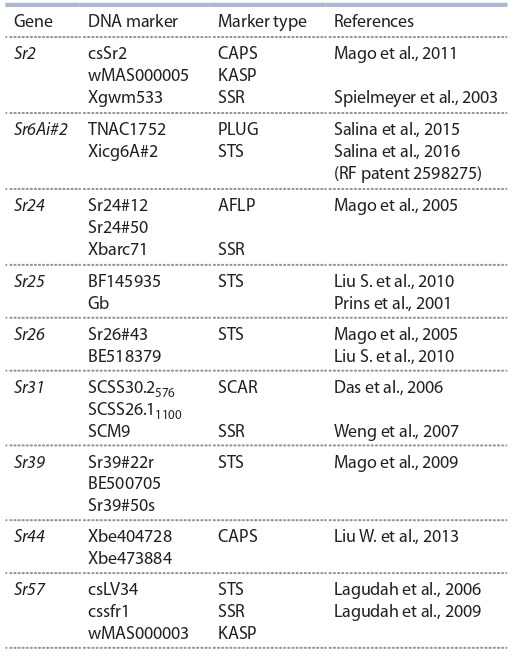
DNA markers for stem rust resistance genes verified on the worldwide wheat germplasm pool Note. AFLP – amplified fragment length polymorphism; CAPS – cleaved amplified polymorphic sequences; KASP – kompetitive allele specific PCR; PLUG – PCR-based landmark unique gene; SCAR – sequence characterized amplified region; SSR – simple sequence repeats; STS – sequence tagged site.

## Заключение

В обзоре сопоставлены результаты исследований популяции возбудителя стеблевой ржавчины с актуальными данными генов устойчивости мягкой пшеницы, эффективными в условиях Западной Сибири. Интерес для опережающей селекции на иммунитет представляют гены устойчивости Sr2, Sr6Ai#2, Sr24, Sr25, Sr26, Sr31, Sr39, Sr40, Sr44, Sr57, для которых показано преобладание авирулентных клонов в местных субпопуляциях гриба. Наиболее популярными при создании пирамидированного генотипа мягкой пшеницы являются гены Sr2, Sr24, Sr31, Sr57, о чем свидетельствует высокий удельный вес сортов и селекционных линий, представленных в международных базах данных, таких как GRIS. Весьма перспективным для селекции на иммунитет является интродуцированный ген Sr6Ai#2, широко представленный в современном отечественном материале. Однако важно подчеркнуть, что после районирования и интенсивного внедрения в производство сорта быстро теряют устойчивость из-за появления новых вирулентных рас патогена. В большинстве случаев широко распространенные коммерческие сорта мягкой пшеницы оказываются восприимчивыми к ржавчине через 7–10 лет (Коваль и др., 2010). Интегральный подход к отбору селекционного материала, включающий данные о генотипах растения-хозяина и патогена, повышает гарантии длительного иммунитета у нового сорта или селекционной линии.

## Conflict of interest

The authors declare no conflict of interest.
